# Blurred Ideas: How Perpetrator Behavior, Target Response, and Observer Gender Can Influence Perceptions of Workplace Sexual Harassment

**DOI:** 10.1177/08862605241271368

**Published:** 2024-08-19

**Authors:** Charlotte Keenan, Courtney von Hippel, Annabelle Neall, Fiona Kate Barlow

**Affiliations:** 1 The University of Queensland, St Lucia, QLD, Australia; 2 Flinders University, Adelaide, SA, Australia

**Keywords:** sexual harassment, in the work place, workplace violence

## Abstract

Despite increasing awareness, sexual harassment remains a significant concern in the workplace. The enduring problem of sexual harassment seems to persist due to a lack of clarity regarding what behaviors qualify as harassment. Furthermore, the interpretation of these behaviors is influenced by contextual and relational factors, contributing to the complexity of addressing and preventing such incidents. This study builds on existing research by investigating how the severity of sexually harassing behavior, the response from the target, and the gender of the participant contribute to labeling behavior as sexual harassment. Using an online experimental scenario-based survey, 1,700 (850 female, 850 male) currently employed participants were presented with a single workplace scenario that manipulated the severity of the sexual harassment behavior and the target’s response. Participants were then asked to assess the appropriateness of the behavior, label it as sexual harassment or not, and rate their confidence in their labeling decision. The results revealed that less severe sexual harassment behaviors, targets who displayed interest, and male participants were more likely to perceive the behavior as less inappropriate and were less inclined to label it as sexual harassment. These findings have implications for shaping the definition of sexual harassment and designing training programs for heightened awareness.

Workplace sexual harassment can be defined as any unwelcome conduct of a sexual nature in the workplace that would offend, humiliate, or intimidate someone (Charlesworth et al., 2011). Academically, there is a clear understanding of how sexual harassment affects the mental well-being of targets (Pina & Gannon, 2012), contributes to toxic workplace cultures (McLaughlin et al., 2017), and damages organizational reputations (Orlitzky et al., 2003). A pertinent gap, however, lies in how laypeople understand sexual harassment, despite the impact of global social movements such as #MeToo (Brittney et al., 2020). In the current scenario-based study we build on past literature (Strom et al., 2023) to investigate whether the type of sexual harassment (e.g., from gender harassment through to sexual imposition) affects how laypersons perceive the behavior. Simultaneously we recognize that sexual harassment is often embedded in complex social relationships—targets of sexual harassment often know, and even have good relationships with, those perpetrating sexual harassment. Thus, we vary the extent to which the target of sexual harassment shows interest in the perpetrators’ behavior and measure the gender of the participant observing the sexual harassment. We aim to build knowledge about how contextual and relational factors affect how people interpret workplace sexual harassment and perceive both perpetrator and target. In doing so we contribute to a literature focused on overcoming common barriers to recognizing and combating sexual harassment at work.

## Labeling of Sexual Harassment

Sexual harassment is a prevalent concern for many individuals, most especially women, younger workers, and members of CALD groups (McDonald, 2012), yet challenges persist in effectively reporting these experiences. The present research focuses on reactions to sexual harassment in adults from the United Kingdom and Australia, where recent data shows high rates of workplace sexual harassment. Specifically, reports suggest that 41% of Australian women in the last 5 years (Australian Human Rights Commission, 2022) and 58% of UK women in their lifetime (Trade Union Congress, 2023) have experienced sexual harassment at work. Both reports also highlight low reporting rates, with only 19% of Australian women and 30% of UK women formally reporting their experiences. These statistics demonstrate how many individuals experience sexual harassment but choose not to report it, often limited by concerns over potential reprisals and being labeled as too sensitive. However, a general societal lack of understanding regarding what behaviors *constitute* sexual harassment (Australian Human Rights Commission, 2022) may influence how and if people see certain behaviors as sexual harassment. Schema theory can help to understand and guide investigations into these factors.

Schemas are mental frameworks that organize prior knowledge and assist with interpreting new situations by allowing the schema holder to make quick inferences about a group or event (Axelrod, 1973). Sexual harassment schemas hold essential information, including definitions, characteristics, and expectations of sexual harassment (Fiske & Taylor, 1991). However, schemas can be influenced by stereotypes and rigid expectations of what sexual harassment should look like. Past quantitative work has shown that the activation of sexual harassment schemas can play a role in whether behaviors are labeled as sexual harassment (Magley & Shupe, 2005). Qualitative research reveals that people have dominant sexual harassment schemas, characterized by explicit sexual harassment behavior, powerful male perpetrators, and vulnerable, yet attractive, female targets (e.g., Popovich et al., 1995). In reality, most sexual harassment is subtle and ambiguous (Sojo et al., 2016), and may therefore go unrecognized due to the inconsistency with sexual harassment schemas. Further qualitative studies reinforce this notion, revealing that people can fail to recognize subtler forms of sexual harassment because they feel that the behavior might reflect miscommunication (Dardis et al., 2021), be unintentional (Nielsen et al., 2024), or not be severe enough to warrant the label of sexual harassment (Bergenfeld et al., 2022; Shupe, 2020).

Reluctance or confusion over labeling sexual harassment can mean the behaviors are not accurately reported, and subsequently not effectively managed (Strom et al., 2023). When sexual harassment goes unreported and unmanaged, it can signal to everyone involved that such behavior is tolerated. This can lead to feelings of institutional betrayal due to the failure of the institution to prevent and respond appropriately (Smith & Freyd, 2014), ultimately increasing the harm to those affected. Thus, to understand what factors can shape how an observer evaluates sexual harassment behavior, experimental scenarios can be used. Scenarios allow for the manipulation of factors to test perceptions that pertain to beliefs and attitudes on topics that can otherwise be difficult to study (Bradbury-Jones et al., 2014). This is particularly beneficial in the case of sexual harassment, where it is not ethical to expose participants to the behavior. Therefore, in the present study we had participants read scenarios where we manipulated the sexual harassment behavior type and level of interest the target shows the perpetrator to understand the influence of these factors on judgments. We also investigated how the gender of the participant may impact perceptions.

## Behavior Type

In line with schema theory, individuals contain expectations and criteria about which behaviors are seen as sexual harassment. A way to understand behaviors that constitute sexual harassment is through Till’s (1980) Classification System, which proposes that sexually harassing behaviors sit on a continuum of increasing severity, ranging from gender harassment (e.g., sexist jokes) to sexual imposition (e.g., fondling) (see Table 1). Past work informs predictions on what behaviors are more likely to be seen as sexual harassment. Research has indicated that behaviors that are more physically imposing (Cummings & Armenta, 2002; Osman, 2004, 2007) or making one’s job contingent on sexual favors (Jacobson & Eaton, 2018) are more likely to activate sexual harassment schemas. Many studies use Fitzgerald et al. (1995) definition of sexual harassment, which proposes there are three types of sexual harassment (gender harassment, unwanted sexual attention, and sexual coercion). While this definition has been seen to be more parsimonious, it collapses sexual bribery and sexual coercion into one behavior as well as seductive behavior and sexual imposition into unwanted sexual attention. Thus, it may not adequately assess the idea that sexual harassment behaviors can differ in perceived severity. Till’s (1980) definition separates these behaviors to allow for the testing of severity. A recent study by Strom et al. (2023) explored different severity types of sexual harassment behaviors and how they affected labeling in experimental scenarios. They found that only 53% of scenarios were correctly labeled by participants, indicating there could be a lack of awareness of what constitutes sexual harassment. In our study, we build upon the work of Strom et al. (2023) by refining their scenarios according to Till’s (1980) classification, to allow for a deeper exploration of behavior severity. Additionally, we adopt a more nuanced approach to labeling by also measuring behavior appropriateness, to examine whether observers perceive the behavior as inappropriate regardless of their labeling decision. As such, we propose that:

**Table 1.** Till’s (1980) Classification System of Sexual Harassment.

Sexual Harassment Type	Definition
Gender harassment	Generalized sexist jokes or remarks
Seductive behavior	Inappropriate and offensive sexual advances
Sexual bribery	Sexual activity solicited by promise of rewards such as promotion
Sexual coercion	Sexual activity required to avoid punishment such as being fired
Sexual imposition	Deliberate touching, fondling, or attempted intercourse

H1: As the type of sexual harassment behavior becomes more severe, the perpetrator’s behavior will be rated as more inappropriate, the behavior will be more likely to be labeled as sexual harassment, and confidence in this labeling decision will be higher, as compared to less severe sexual harassment behaviors.

## Target Response

In this study we not only varied the type of sexual harassment but also whether the target felt romantic or sexual interest in the perpetrator of sexual harassment. To the best of our knowledge, ours is the first work to explicitly look at target interest, but substantial work looks at related ways in which targets respond to sexual harassment. For example, work has found that sexual harassment is most readily perceived when the target responds consistently, assertively, and discouragingly to the perpetrator (Yagil et al., 2006, see also Mills & Scudder, 2023). Related work has found sexual harassment is more likely to be correctly labeled if the target physically or verbally resists the behavior (as opposed to not resisting) (Osman, 2007; Randall, 2010). Osman (2004) also found that when a target’s response to sexual harassment was to say “stop” while smiling, instead of frowning or not showing a facial expression, people were less likely to identify sexual harassment.

In the real world, however, it is not just a case of whether a target of sexual harassment responds negatively. Sexual harassment often takes place between colleagues with their own histories of friendship, and sometimes even romantic interest (Pierce & Aguinis, 2001). Those observing sexual harassment will be watching both parties, not just the perpetrator. In the present work, we acknowledge this messiness by varying whether the target of sexual harassment is in fact receptive to the behavior. We propose that target interest may reduce the likelihood that observers label workplace sexual harassment as such, as it should contradict their sexual harassment schemas. Specifically, past work has found that common schemas around sexual harassment prescribe strong resistance as necessary (Osman, 2004); sexual or romantic interest, or receptivity to sexual harassment, clearly violates the assumption of outraged resistance. Further, interest may contradict participants’ understanding of sexual harassment as an inherently unwanted behavior. Thus, we would expect that:

H2: When the target does not show interest toward the perpetrator’s actions, the perpetrator’s behavior will be rated as more inappropriate, the behavior will be more likely to be labeled as sexual harassment, and confidence in this labeling decision will be higher, as compared to when the target appears interested in the perpetrator’s actions.

In considering our prediction, however, the type of sexual harassment perpetrated must be accounted for. Classic schemas about sexual harassment see sexual harassment as explicit and severe. In Till’s classification system, this would include behaviors such as sexual bribery, coercion, or imposition. Given how clearly defined and explicit these behaviors are we propose that target interest (or lack thereof) might not be as impactful as it is when the sexual harassment type is more subtle, and hence ambiguous (e.g., sexist remarks in the context of interest might be perceived as friendly office banter). We propose that:

H3: The effect of target response on the three outcome variables will be moderated by behavior type. Specifically, the relationship between target response and the outcome variables will be stronger at lower levels of behavior type, such that when the target is interested and the behavior type is less severe (i.e., gender harassment) the behavior will be rated as significantly less inappropriate, compared to when the target is not interested and the behavior type is more severe (i.e., sexual imposition).

## Character Judgments

Thus far, many studies have focused on sexual harassment perceptions based on the perpetrator’s actions, often overlooking the potential influence of other people, such as the target, in shaping judgments. The previous research has focused on the perpetrator’s actions by measuring likelihood of sexually harassing the target or how guilty the perpetrator is (Dinh et al., 2022; Kessler et al., 2020; Strom et al., 2023). While this provides useful information, it neglects to account for how observers view the target’s actions as well as the interaction between those involved. Some studies have explored perceptions of the target, including factors such as perceived credibility that can influence victim blaming and beliefs around false accusations (Balogh et al., 2003; Bhattacharya & Stockdale, 2016; De Judicibus & McCabe, 2001). However, to the best of our knowledge, only limited studies have explored evaluations of both the target and perpetrator simultaneously (Bursik & Gefter, 2011). For example, in a scenario-based study, Pica et al. (2020) manipulated prior allegations from the target to assess the impact on both target and perpetrator’s credibility. They found prior target allegations hurt the credibility of the target more than the perpetrator (Pica et al., 2023). The results of this study support our assertion that characteristics of a sexual harassment incident might simultaneously affect evaluations of both perpetrator and target. This could even be particularly the case in workplace contexts where there are strict expectations for professional conduct (Zelin et al., 2022). As noted earlier, targets tend to be believed most when they resist sexual harassment in clear and concrete terms. Further, perpetrators seem to be most clearly blamed when the type of sexual harassment is overt and stereotypical. Therefore, it is conceivable that participants may see the target’s actions as least appropriate when they are interested and/or are the target of gender harassment or other less severe behavior types. However, we do not make a firm prediction and leave these analyses as exploratory.

## Observer Gender

In addition to situational factors influencing the inclination to label an incident as sexual harassment, the gender of the observer can also impact perceptions. Numerous studies indicate that women generally exhibit a higher likelihood of accurately identifying sexual harassment, recognizing ambiguous behavior as sexual harassment, considering a broader range of behaviors as meeting the definition of sexual harassment, and being less inclined to blame the target for the event (Rothgerber et al., 2021; Rotundo et al., 2001; Shechory Bitton & Ben Shaul, 2013). One potential explanation for this pattern is that men tend to attribute sexual desire to women more frequently and perceive women as behaving more sexually in interactions that are inherently friendly in nature (Lindgren et al., 2008; Osman, 2004). As such, we expect:

H4: Male participants will rate the behaviors as more appropriate, and the behaviors will be less likely to be labeled as sexual harassment, as compared to female participants.

## The Current Study

To reiterate, in this study participants are presented with scenarios where we manipulate sexual harassment type and target response. Our aim is to investigate how these factors influence perceptions of perpetrator and target behavior appropriateness, labeling of sexual harassment, and confidence in the labeling decision. We aim to build on the work of Strom et al. (2023) and offer insights into why labeling of sexual harassment may be low. Specifically, we will explore whether participants still perceive behavior as inappropriate regardless of whether it is labeled as sexual harassment.

The materials for this pre-registered study are available in the Open Science Framework: https://bit.ly/44V43DH.^1^ Research data is available upon reasonable request from the corresponding author.

## Method

### Sample

Participants (*N* = 1,700) were recruited via Prolific (The University of Queensland Research Ethics ID: #2022/HE000966). Eligible participants were over the age of 18 and employed in Australia (5.1%) or the United Kingdom (94.9%). The sample comprised 850 men and 850 women with a mean age of 39.44 (standard deviation [*SD*] = 11.77). Most participants identified as White/Caucasian (85.0%), held an undergraduate degree or higher (60.8%), and were employed full-time (73.9%). The average tenure in their current organization was 7.39 years (*SD* = 7.32), with 40.7% working in organizations with more than 1000 employees. All job levels were represented, with 25.7% working at the middle level. Participants worked across diverse industries, with education and training being the highest reported employment sector (12.5%). While 71.9% of participants reported no workplace sexual harassment experience, 22.7% did. Finally, 61.4% reported not receiving workplace sexual harassment training.

### Procedure

Participants completed a voluntary online Qualtrics survey framed as “interpersonal conflict in the workplace.” The study was between groups 5 (sexual harassment type: gender harassment, seductive behavior, sexual bribery, sexual coercion, and sexual imposition) × 2 (target response: interested, not interested) × 2 (participant gender: male, female) design. Participants were randomly assigned to one scenario out of a possible 10. They were asked to read through the scenario and then indicate how appropriate the behavior was of the perpetrator, whether the behavior was sexual harassment, and how confident they were in that latter decision. Following this, they were asked to indicate how appropriately they felt that the target behaved. They were compensated for their time according to Prolific set rates.

## Measures

### Sexual Harassment Scenarios

Ten scenarios were developed by modifying those from Strom et al. (2023) to align with Till’s (1980) five-category classification system. The scenarios were manipulated to depict one of five types of sexual harassment behavior (gender harassment, seductive behavior, sexual bribery, sexual coercion, and sexual imposition). The scenarios were also manipulated to vary the target’s response, indicating whether she seemed interested or not interested. The scenarios were informally piloted with a small sample from an introductory psychology student pool to ensure they accurately depicted the type and severity level. The full list of scenarios can be found in the OSF. Example scenarios for the gender harassment not-interested and interested conditions, respectively, are below.

Emma is a strategy analyst at a large consulting firm and is responsible for giving biweekly presentations to her coworkers. During and after her presentation, multiple men from the office commented on how great her figure looked in her dress. Her boss, Patrick, is present and nods along with her male coworkers’ comments. Emma is offended and thinks the dress is a poor choice.

Emma is a strategy analyst at a large consulting firm and is responsible for giving biweekly presentations to her coworkers. During and after her presentation, multiple men from the office commented on how great her figure looked in her dress. Her boss, Patrick, is present and nods along with her male coworkers’ comments. Emma is flattered and thinks the dress is a good choice.

### Behavior Appropriateness

As per our focus on perceived appropriateness, we created items that asked participants to rate behavior appropriateness of both the perpetrator and target in the scenario (e.g., “How appropriate was (perpetrator’s) behavior?”) on a 7-point Likert-type scale (1 = *completely inappropriate*, 7 = *completely appropriate*).

### Sexual Harassment Labeling

In line with previous research (Strom et al., 2023), we measured the propensity of participants to label the behavior of the perpetrator as sexual harassment using a dichotomous scale of yes or no, for example, “Is (perpetrator’s) behavior sexual harassment?”

### Confidence in Sexual Harassment Labeling Decision

Consistent with other research (Balogh et al., 2003), we measured confidence in the labeling decision via the following question: “How confident are you it is/is not sexual harassment?” on a 5-point scale (1 = *not at all confident*, 5 = *completely confident*).

### Control Variables

Responses to sexual harassment can vary due to work and personal characteristics as well as individual experiences, which in turn shape whether behavior is labeled as sexual harassment (Stockdale et al., 1995). Thus, we measured work characteristics such as job level, employment sector, and tenure, as research indicates that lower-status employees and those working in male-dominated spaces are more likely to experience sexual harassment (Pina & Gannon, 2012). We also measured demographics such as age and ethnicity, as employees who are younger and not white are more vulnerable to sexual harassment (McDonald, 2012). Finally, we assessed sexual harassment awareness (e.g., experience and training).

### Analytical Approach

To investigate whether there were main effects and interactions for sexual harassment type, target response, and participant gender on perceived behavior appropriateness and confidence in labeling we conducted between-subject analyses of variance (ANOVAs). Bonferroni adjusted pairwise comparisons were used to follow up any main effects of sexual harassment type (given its five levels) and any interactions between predictor variables. To test whether sexual harassment type, target response, and participant gender, influenced sexual harassment labeling we conducted a binary logistic regression. All analyses were run with and without control variables included, as described below.

## Results

### Preliminary Analyses

Data underwent outlier screening, comparative analyses, and missing data testing. We conducted the main analyses with and without control variables included. The primary pattern of effects was substantively the same regardless of whether the control variables were in the model. For ease of interpretation, we therefore present our results without control variables included, although we note any small changes where they exist. In general, our control variables (including tenure, company size, employment status, ethnicity, employment sector (male vs. female dominated), workgroup gender ratio, country of residence, and age) were not associated with our outcome variables. There were, however, a few exceptions. Those who had direct experience of sexual harassment or had undergone training were more confident in their decision around whether to label the observed behavior as sexual harassment (or not). Finally, people in more senior relative to junior positions tended to rate perpetrator (but not target) behavior as less inappropriate. They were also more confident in their sexual harassment labeling decision. The full results of our analyses with control variables included are in the supplementary online material, along with a post hoc power analysis and a full breakdown of results per condition.

### Perpetrator Behavior Appropriateness

For model statistics and means per condition refer to Table 2. Results revealed main effects of target response, sexual harassment type, and participant gender. Participants saw the perpetrator as less inappropriate when the target was interested relative to uninterested, and men generally rated the perpetrator as less inappropriate than did women. Turning to type, gender harassment and seductive behavior were seen as the least inappropriate, and equally so (*p* = .861). Each was rated as less inappropriate than sexual bribery, sexual coercion, and sexual imposition (*ps* < .001). There were no differences in ratings of inappropriateness of the latter three types (*ps* > .05).

**Table 2.** ANOVA Statistics for Response, Type, and Gender on Perpetrator Behavior Appropriateness.

	*M*	*SD*	*df*	*F*	*p*	Partial η^2^
Corrected model			1, 1,679	23.15**	<.001	.21
Response			1, 1,679	53.41**	<.001	.03
Interested	1.53	1.12				
Not interested	1.22	0.82				
Type			4, 1,679	69.37**	<.001	.14
Gender harassment	1.82	1.27				
Seductive behavior	1.81	1.35				
Sexual bribery	1.07	0.56				
Sexual coercion	1.05	0.43				
Sexual imposition	1.13	0.61				
Gender			1, 1,679	23.51**	<.001	.01
Male	1.48	1.13				
Female	1.27	0.82				
Response × Type			4, 1,679	19.07**	<.001	.04
Response × Gender			1, 1,679	1.22	.270	.00
Type × Gender			4, 1,679	1.89	.109	.00
Response × Type × Gender			4, 1,679	0.37	.831	.00

*Note*. ANOVA = analysis of variance; *SD* = standard deviation.

Signficance levels: * *p* < .05. **p < .001.

**Figure 1.**
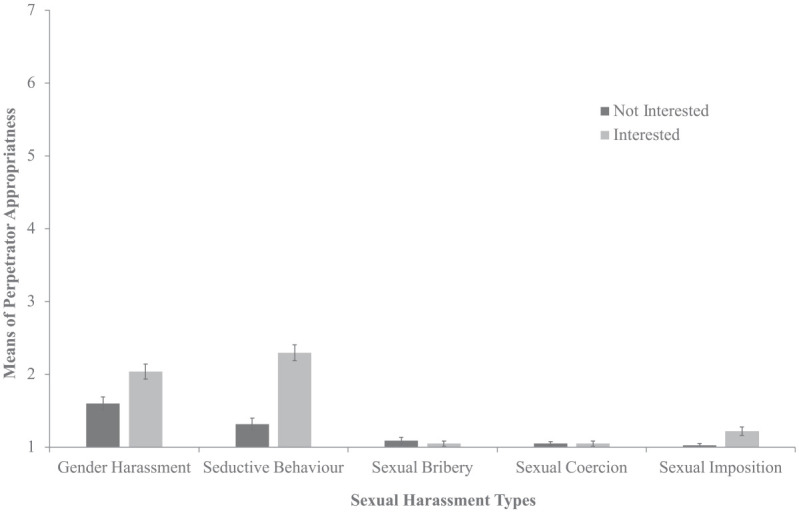
Sexual harassment type and target response on perpetrator behavior appropriateness. *Note*. Error bars represent standard errors.

As seen in Figure 1, the main effects of both response and type were qualified by an interaction between the two. Target interest versus disinterest resulted in ratings of less perpetrator inappropriateness only when the perpetrator engaged in gender harassment (*p* < .001), seductive behavior (*p* < .001), or sexual imposition (*p* = .046). No interactions were found between gender and response, or type. However, an interaction did emerge in the control analysis for gender and type, where male participants rated the less severe types are less inappropriate than female participants. Additionally, participants with higher job levels rated the behavior as less inappropriate. No three-way interaction was found.

### Sexual Harassment Labeling

**Figure 2.**
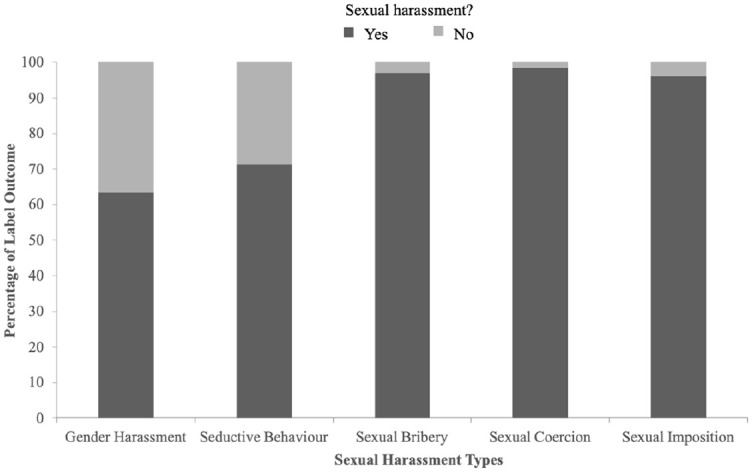
Labeling rates for sexual harassment behaviors.

Direct binary logistic regression analyzed the influence of sexual harassment type, target response, and participant gender on labeling behavior as sexual harassment. The model, with these three variables, was statistically significant χ^2^(6, 1,700) = 395.62, *p* < .001, indicating it could differentiate between participants who labeled each behavior as sexual harassment and those who did not. The results aligned closely with appropriateness ratings (see Table 3). More severe types (particularly sexual coercion) had higher odds of being labeled sexual harassment (see Figure 2). However, in the control analysis, seductive behavior did not increase the odds of labeling. Finally, female participants or those exposed to the not-interested condition were more likely to label each behavior as sexual harassment (vs. not).

**Table 3.** Coefficients of the Sexual Harassment Labeling Model.

							95% CI for OR	
Variables		β	*SE*	Wald	*p*	OR	Lower	Upper
Gender (Female)								
Male		−0.60	.16	14.10	<.001	0.55	0.40	0.75
Type (gender harassment)								
Seductive behavior		0.40	.17	5.25	.022	1.49	1.06	2.09
Sexual bribery		3.11	.35	80.48	<.001	22.39	11.35	44.16
Sexual coercion		3.82	.47	66.12	<.001	45.38	18.09	113.82
Sexual imposition		2.84	.31	83.20	<.001	17.12	9.30	31.51
Response (not interested)								
Interested		−1.35	.17	65.45	<.001	0.26	0.19	0.36
Constant		1.61	.18	79.08	<.001	4.98		

*Note*. The reference category for each variable is indicated in the parentheses next to the variable name. OR = odds ratio; CI = confidence interval; *SE* = standard error.

### Confidence in Sexual Harassment Labeling Decision

For model statistics and means per condition refer to Table 4. Results showed the predicted main effects for target response and sexual harassment type, but not participant gender. Labeling confidence was higher when the target was not interested compared to when interested. For type, gender harassment and seductive behavior differed with each other (*p* = .034), as well as with the other three more severe types (*p*s < .001). The three most severe types did not differ among each other (*p*s > .05). This finding suggests higher confidence in labeling with more severe types of sexual harassment.

**Table 4.** ANOVA Statistics for Response, Type, and Gender on Confidence in Sexual Harassment Labeling Decision.

	*M*	*SD*	*df*	*F*	*p*	Partial η^2^
Corrected model			1, 1,667	26.60**	<.001	.23
Response			1, 1,667	75.81**	<.001	.04
Interested	4.11	1.09				
Not interested	4.49	0.88				
Type			4, 1,667	96.09**	<.001	.19
Gender harassment	3.69	1.17				
Seductive behavior	3.89	1.07				
Sexual bribery	4.67	0.68				
Sexual coercion	4.68	0.68				
Sexual imposition	4.59	0.86				
Gender			1, 1,667	0.89	.345	.00
Male	4.28	1.00				
Female	4.32	1.02				
Response × Type			4, 1,667	8.65**	<.001	.02
Response × Gender			1, 1,667	0.00	.974	.00
Type × Gender			4, 1667	1.10	.357	.00
Response × Type × Gender			4, 1667	1.00	.408	.00

*Note*. ANOVA = analysis of variance; *SD* = standard deviation

Signficance levels: * *p* < .05. **p < .001.

**Figure 3.**
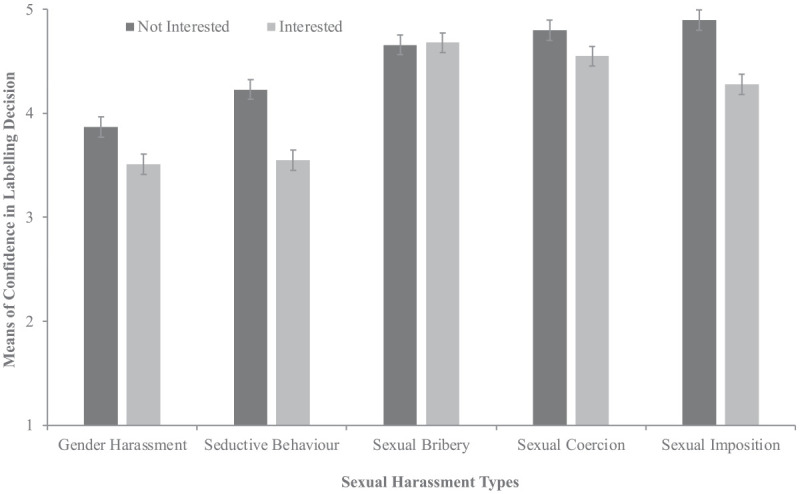
Sexual harassment type and target response on confidence in sexual harassment labeling decision. *Note*. Error bars represent standard errors.

As seen in Figure 3, the main effects of response and type were qualified by an interaction between the two. Target interest versus disinterest resulted in lower labeling confidence with gender harassment (*p* < .001), seductive behavior (*p* < .001), sexual coercion (*p* = .012), and sexual imposition (*p* < .001). Target interest had a greater impact on confidence when the type was less severe, however, there were effects for interest with the two most severe types. No interactions were found between genders with response or type. There was no three-way interaction. In the control analysis, participants who had experienced sexual harassment received workplace training on it and were higher in their job level, and had more confidence in their labeling decision.

### Target Behavior Appropriateness

For model statistics and means per condition refer to Table 5. Results revealed main effects for target response and sexual harassment type, but not participant gender. The target’s behavior was rated as more inappropriate when the target was interested versus not. Regarding type, the target’s behavior was rated as less appropriate across the less severe types (gender harassment and seductive behavior) compared to the other types (*p*s < .001). No differences were found among the three most severe types (*p*s > .05).

**Table 5.** ANOVA Statistics for Response, Type, and Gender on Target Behavior Appropriateness.

	*M*	*SD*	*df*	*F*	*p*	Partial η^2^
Corrected model			1, 1,679	156.04**	<.001	.64
Response			1, 1,679	2,245.70**	<.001	.57
Interested	3.21	1.98				
Not interested	6.38	1.22				
Type			4, 1,679	53.78**	<.001	.11
Gender harassment	5.09	1.75				
Seductive behavior	5.62	1.59				
Sexual bribery	4.43	2.60				
Sexual coercion	4.29	2.37				
Sexual imposition	4.54	2.64				
Gender			1, 1,679	0.38	.540	.00
Male	4.81	2.20				
Female	4.78	2.37				
Response × Type			4, 1,679	121.14**	<.001	.22
Response × Gender			1, 1,679	5.56*	.019	.00
Type × Gender			4, 1,679	2.51*	.040	.00
Response × Type × Gender			4, 1,679	1.76	.134	.00

*Note*. ANOVA = analysis of variance; *SD* = standard deviation

* *p* < .05. **p < .001.

**Figure 4.**
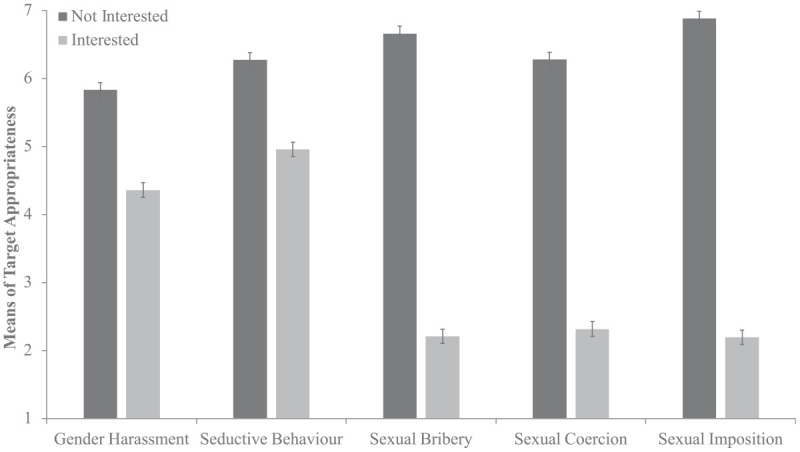
Sexual harassment type and target response on target behavior appropriateness. *Note*. Error bars represent standard errors.

As seen in Figure 4, an interaction between response and type emerged such that target behavior was rated as less appropriate when the target showed interest (*p* < .001). Unexpectedly, this effect was greater for the more severe types compared to the less severe types (*p* < .001) An interaction between gender and response was also found, where larger differences were found between the interested and not-interested conditions for female participants (*p* < .001). An interaction between gender and type emerged, where female participants rated the behavior in sexual imposition as more inappropriate than males (*p* = .039). However, this interaction should be interpreted with caution as it was not present in the control analysis. No three-way interaction was observed.

## Discussion

Sexual harassment is a widespread issue impacting workforce productivity, morale, and safety; recognizing and accurately labeling the behavior is crucial for managing its detrimental effects. Therefore, this study aimed to explore factors that hinder the recognition of sexual harassment. The research addresses knowledge gaps by offering an explanation for why individuals may struggle to label sexual harassment, while also examining other factors that could interfere with recognizing such behavior. This study furthers our understanding of why people struggle labeling sexual harassment, which can inform ways to improve reporting systems and develop evidence-based interventions.

Most hypotheses were supported and aligned with existing research. The first hypothesis, which predicted more severe types of sexual harassment would lead to higher ratings of inappropriateness, increased likelihood of labeling, and greater labeling confidence, was supported and is consistent with previous literature (del Carmen Herrera et al., 2017; McCarty et al., 2014). Similarly, the second hypothesis, suggesting that when the target shows no interest in the perpetrator, the behavior is perceived as more inappropriate, is more likely to be labeled as sexual harassment, and there is greater labeling confidence, was supported in line with similar research (Osman, 2004; Yagil et al., 2006). The third prediction, which proposed participants would rate the perpetrators’ behavior as less inappropriate when the sexual harassment types were less severe, was mostly supported. This finding generally aligns with quantitative (Weiner et al., 2010) and qualitative (Bergenfeld et al., 2022) work which finds participants are more likely to view a case as sexual violence if the behavior is severe and the target is aggressive, rather than submissive. However, an unexpected effect emerged where perpetrator behavior was rated as less inappropriate for the most severe type with target interest. While this pattern should not be overinterpreted, it aligns with a general trend within the data whereby regardless of the target response, sexual bribery and coercion stand out as clearly inappropriate and consistently labeled as sexual harassment. The fourth prediction, proposing men would perceive the perpetrator’s behavior as more appropriate and be less likely to label it as sexual harassment compared to women, was supported, reflecting gender differences found in prior research (Rotundo et al., 2001). Lastly, exploratory analysis revealed that target behavior was rated as less appropriate for more severe types or if the target showed interest.

### Theoretical Implications

These findings yield three key theoretical implications. Notably, the similarity in appropriateness as well as labeling ratings between sexual bribery (i.e., solicitation of sexual activity by promise of rewards) and sexual coercion (i.e., demanding sexual favors to avoid punishment) suggests they could be grouped together as per the Fitzgerald et al. (1995) definition. Despite Till’s (1980) definition proposing the behaviors to be distinct, it is possible that both researchers and participants do not practically differentiate between the act of offering a reward for sex (bribery) and threatening punishment for failing to have sex (coercion). It is possible that both acts are inherently seen as coercive as they concern target’s livelihoods and make workplace success contingent on sexual acts. In both cases, the power differential is explicitly highlighted and exploited, in line with schemas around sexual harassment (Magley & Shupe, 2005). It is possible that sexual imposition, when coupled with sexual interest from the target, fits less neatly into the schema. Future work should explore these possibilities.

Moving on from debates around bribery and coercion, in general, and consistent with the literature (del Carmen Herrera et al., 2017; McCarty et al., 2014), the findings indicate that more severe types are seen as more representative of sexual harassment. This finding has implications for the less severe but more prevalent type of gender harassment (Leskinen et al., 2011), which may not be easily recognized as sexual harassment despite having long-term effects that can be just as damaging as the severe types (Sojo et al., 2016). It may be that participants in the present study are adhering to outdated schemas that reinforce stereotypical expectations of severe and predatory sexual harassment behaviors. However, these stereotypes may not accurately reflect the broad spectrum of sexual harassment behaviors experienced by targets.

Moreover, it seems that participants hold schematic expectations that targets should not respond in ways that might indicate interest. This is a problem as people cope with problems in lots of different ways, including trying to address the situation directly or trying to reduce the emotional impact of the situation (Lazarus & Folkman, 1984). Of relevance is passive emotion-focused coping, where targets can use strategies that include wishful thinking, denial, or “saving face” to get through difficult situations (Scarduzio et al., 2018). In workplaces, where maintaining harmony is crucial for job security and relationships, passive emotion-focused responses may be common from targets, even though observers potentially struggle interpreting a target appearing to comply with a perpetrator. As such, target interest likely sits uneasily with traditional understandings of sexual harassment. Observers may not know what to do when the target is going along with the perpetrator and could blame the target for not displaying a clear distressed response, as dictated by the prevailing schema. Interestingly, in the case of the most severe type of sexual harassment, a noteworthy difference emerged between the interested and not-interested conditions. This contrast was unexpected at this severity level, as it was presumed that the severity of the behavior should negate the effect of target interest. However, this discrepancy might be linked to sexual consent schemas, where compliance with the behavior is interpreted as consent, especially when individuals do not explicitly express a refusal (Stuart et al., 2019).

Interestingly, the schematic expectations for targets not to display any interest in the most severe types of sexual harassment appear counterintuitive. Given the heightened level of threat in such situations, it may be particularly challenging for the target to convey disinterest. This paradox could potentially be explained by the well-established phenomenon of rape myths, which are unfounded and harmful beliefs about sexual assault and rape (Burt, 1980, p. 217). These myths suggest that targets must unequivocally resist all behaviors and provide clear evidence of such resistance. Consequently, if targets appear seductive or too permissive of the behavior, they are often unjustly labeled as “asking for it” and condemned. This situation may be exacerbated in instances of sexual harassment where severe behavior is perceived as less ambiguous and more clearly wrong. This puts the responsibility on targets to behave in ways that demonstrate the perpetrator's misconduct is inappropriate; despite the heightened risk of the behavior, which may include an increased concern of escalating sexual violence or threats to job security (Knapp et al., 2019). Thus, the target may not deem it safe to express concern or outrage.

This study also identified gender differences in perceptions of sexual harassment. Gendered hierarchies and behaviors still persist in workplaces, where women are more likely to recognize the power imbalances inherent in sexual harassment compared to men (Wamoyi et al., 2022). Power is not only gendered, however, it is also racialized. Women of color are disproportionally affected by sexual harassment (Armstrong et al., 2018) and can face a double jeopardy of sexual and racial discrimination (Crenshaw, 1989). An intersectional approach to understanding sexual harassment positions race and gender as factors that affect people, not only additively, but also in unique combinations (Crenshaw, 1991). Black women in the United States, for example, contend not only with sexism and racism but also sexualized stereotypes connected to slavery (including sexual slavery; Crenshaw, 1991). Recent qualitative work finds that Black women report feeling sexualized, stereotyped as promiscuous, and in some cases fetishized (Rosenthal et al., 2020; Spates et al., 2020). Practically, Black women in the U.S. earn less and experience more job precarity than White women, and in many cases have to work multiple jobs or extra shifts (for an overview refer to Spates et al., 2020). Such factors might jointly work to increase the extent to which Black women face sexual harassment and the extent to which they feel safe to report it. Recent research by Hart (2023) demonstrates that Black women are seen as less credible targets of sexual harassment due to not fitting the rare “archetypal” sexual harassment script (see Armstrong et al., 2018, for a review). We note that sexual harassment experiences will also be affected by other identities including those around gender, and sexuality. There have been recent calls, for example, to take an intersectional approach to focus on understanding how Black Queer women experience sexual harassment (Brassel et al., 2020). We echo these calls and suggest that future work extending ours should be conducted in which first race, and then perhaps sexual or gender identity of both victim and perpetrator are varied. Similarly, it will be important to look at how people’s own identities on these dimensions interact to predict responses to sexual harassment.

### Practical Implications

The theoretical implications discussed have practical consequences for workplace management. First, if individuals cannot recognize a behavior as sexual harassment, they may encounter challenges reporting it (Goh et al., 2022). Without reporting, management will remain unaware of the behavior, leading to a failure to implement measures to address and prevent future incidents. This lack of intervention could potentially result in the escalation of sexual harassment in terms of severity, frequency, and occurrence (Knapp et al., 2019). Additionally, work systems that make it difficult to recognize and report sexual harassment can signal to targets, perpetrators, and members of the organization that the behavior is tolerated (Smith & Freyd, 2014), leading employees to feel betrayed by their institution. In general, institutional betrayal can result in psychological (e.g., emotional and physical distress) and pragmatic (e.g., inequality) harm for affected parties. A further consequence is desensitization to sexual harassment behaviors as they become normalized. Desensitization can then shape the ability to recognize the behavior, as research has found that in industries with high rates of sexual harassment, employees are less likely to recognize the behavior (Strom et al., 2023). Therefore, the study shows the importance for workplaces to consider implementing awareness and education programs to enhance understanding of sexual harassment. This is especially important as approximately 61% of participants in the current study reported not having received sexual harassment training in the workplace, indicating a clear opportunity for targeted information dissemination moving forward.

### Strengths, Limitations, and Future Directions

This study has demonstrated variations in how individuals interpret sexual harassment behavior, revealing potential challenges in workplace identification. There appear to be many factors that can influence perceptions, leading to differences in whether a behavior is considered appropriate or labeled as sexual harassment. Notably, gender harassment emerged as a type of sexual harassment that was perceived as less inappropriate and less likely to be labeled as sexual harassment. These findings contribute to the existing literature by experimentally demonstrating that layperson definitions may not align with legal or academic definitions of sexual harassment (Charlesworth et al., 2011). Furthermore, the large sample size in the study was able to show gender differences in perceptions of sexual harassment behavior.

Due to the use of single-item measures, concerns about reliability and validity arise (Allen et al., 2022). Nevertheless, there is a growing body of evidence supporting the validity and reliability of single-item measures, challenging traditional multi-item approaches (Matthews et al., 2022). The use of fictionalized scenarios might not fully capture the complexity of real-life observer experiences in sexual harassment incidents, especially in workplace settings with preexisting opinions and historical context. However, scenarios are widely employed and valuable for understanding schematic interpretations of events (Erfanian et al., 2020).

It is necessary to note the participant’s background, as residents of the United Kingdom and Australia may interpret sexual harassment behaviors differently than individuals in other countries. Research on desensitization and cultural differences has shown variations, especially in non-Western, Educated, Industrial, Rich, and Democratic countries (Mishra & Davison, 2020) This difference is particularly notable in countries with high power distances (e.g., Brazil), where individuals are less likely to label certain behaviors as sexual harassment compared to countries with low power distances (e.g., United States; Pryor et al., 1997). More proximal environmental factors may also have an impact, such as whether participants have direct experience with sexual harassment or sexual harassment training, or whether people’s particular workplaces are female versus male dominated. While our analyses of control variables provide inconsistent support for these possibilities, studies with more robust measures or complex breakdowns of industry, for example, may find a meaningful effect on how sexual harassment is understood (as per Magley & Shupe, 2005 and Strom et al., 2023). Future research should consider how characteristics of the workplace, person, and sexual harassment incident jointly affect how and whether people understand workplace behaviors as sexual harassment.

Given that gender harassment was less frequently labeled as sexual harassment and perceived as more appropriate in this study, it is crucial to further investigate this most common type of sexual harassment (Leskinen et al., 2011). Future studies should aim to enhance awareness of gender harassment, exploring whether the challenge lies in defining it or if other mechanisms contribute to its underrecognition. Incorporating organizational factors and examining the interaction between people and their environment would provide valuable insights into how people perceive the contextual aspects of such incidents. Furthermore, future research should consider national cultural differences in the interpretation of sexual harassment within workplace settings.

## Conclusion

In summary, sexual harassment is more complex than commonly believed. This study highlights potentially persistent schemas that reflect stereotypical expectations of what constitutes sexual harassment while overlooking all the diverse types and responses to such behaviors. Several factors appear to influence judgments of sexual harassment behaviors, including severity, the target’s reaction, and the observers’ gender. It is important to continue uncovering barriers that can hinder workplace sexual harassment labeling, so we can develop effective prevention strategies for the future.

## Supplemental Material

sj-docx-1-jiv-10.1177_08862605241271368 – Supplemental material for Blurred Ideas: How Perpetrator Behavior, Target Response, and Observer Gender Can Influence Perceptions of Workplace Sexual Harassment Supplemental material, sj-docx-1-jiv-10.1177_08862605241271368 for Blurred Ideas: How Perpetrator Behavior, Target Response, and Observer Gender Can Influence Perceptions of Workplace Sexual Harassment by Charlotte Keenan, Courtney von Hippel, Annabelle Neall and Fiona Kate Barlow in Journal of Interpersonal Violence

## Notes

1. Additional variables were explored as a part of the larger study.
