# Supplementary material for: Blurred Ideas: How Perpetrator Behavior, Target Response, and Observer Gender Can Influence Perceptions of Workplace Sexual Harassment
Source: J Interpers Violence. 2024 Aug 19;40(13-14):3132–61. doi: 10.1177/08862605241271368 (PMC12130594; doi:10.1177/08862605241271368)
Supplement: sj-docx-1-jiv-10.1177_08862605241271368 – Supplemental material for Blurred Ideas: How Perpetrator Behavior, Target Response, and Observer Gender Can Influence Perceptions of Workplace Sexual Harassment [file sj-docx-1-jiv-10.1177_08862605241271368.docx]

**SOM Table 01**

| Independent Variables | | | Perpetrator Behavior Appropriateness | | Labeling Frequencies (% and *n*) | | Confidence in Labeling Decision | | Target Behavior Appropriateness | |
| --- | --- | --- | --- | --- | --- | --- | --- | --- | --- | --- |
| Target Response | Behavior Type | Participant Gender | *M* | *SD* | *Yes* | *No* | *M* | *SD* | *M* | *SD* |
| Interested | Gender Harassment | Male | 2.25 | 1.42 | 49.4% (42) | 50.6% (43) | 3.52 | 1.14 | 4.38 | 1.55 |
|  |  | Female | 1.84 | 1.22 | 62.4% (53) | 37.6% (32) | 3.51 | 1.18 | 4.34 | 1.67 |
|  | Seductive Behavior | Male | 2.51 | 1.48 | 41.2% (35) | 58.8% (50) | 3.53 | 1.02 | 4.75 | 1.57 |
|  |  | Female | 2.09 | 1.34 | 58.1% (50) | 41.9% (36) | 3.58 | 1.19 | 5.16 | 1.65 |
|  | Sexual Bribery | Male | 1.09 | 0.63 | 96.5% (82) | 3.5% (3) | 4.67 | 0.63 | 2.29 | 1.57 |
|  |  | Female | 1.01 | 0.11 | 98.8% (84) | 1.2% (1) | 4.69 | 0.70 | 2.13 | 1.60 |
|  | Sexual Coercion | Male | 1.09 | 0.67 | 96.5% (82) | 2.4% (2) | 4.54 | 0.80 | 2.58 | 1.42 |
|  |  | Female | 1.00 | 0.00 | 98.8% (83) | 1.2% (1) | 4.57 | 0.75 | 2.06 | 1.34 |
|  | Sexual Imposition | Male | 1.36 | 1.25 | 88.4% (76) | 11.6% (10) | 4.22 | 1.11 | 2.55 | 1.78 |
|  |  | Female | 1.08 | 0.39 | 96.5% (82) | 2.4% (2) | 4.34 | 1.00 | 1.86 | 1.41 |
| Not Interested | Gender Harassment | Male | 1.72 | 1.19 | 65.9% (56) | 34.1% (29) | 3.86 | 1.11 | 5.73 | 1.61 |
|  |  | Female | 1.48 | 1.12 | 76.5% (65) | 23.5% (20) | 3.88 | 1.20 | 5.93 | 1.52 |
|  | Seductive Behavior | Male | 1.48 | 1.31 | 91.9% (79) | 8.1% (7) | 4.05 | 1.02 | 6.22 | 1.21 |
|  |  | Female | 1.15 | 0.73 | 94.1% (80) | 5.9% (5) | 4.42 | 0.80 | 6.33 | 1.30 |
|  | Sexual Bribery | Male | 1.18 | 0.91 | 95.2% (80) | 3.6% (3) | 4.72 | 0.53 | 6.56 | 1.20 |
|  |  | Female | 1.01 | 0.11 | 96.5% (82) | 3.5% (3) | 4.60 | 0.83 | 6.75 | 0.83 |
|  | Sexual Coercion | Male | 1.06 | 0.45 | 98.8% (83) | 1.2% (1) | 4.82 | 0.54 | 6.27 | 1.19 |
|  |  | Female | 1.04 | 0.33 | 98.8% (83) | 1.2% (1) | 4.77 | 0.55 | 6.29 | 1.24 |
|  | Sexual Imposition | Male | 1.06 | 0.45 | 98.8% (84) | 1.2% (1) | 4.91 | 0.37 | 6.85 | 0.57 |
|  |  | Female | 1.00 | 0.00 | 100% (86) | 0% (0) | 4.90 | 0.49 | 6.92 | 0.39 |

*Descriptive Statistics for Key Outcome Variables*

*Note.* A total of three missing cases.

*Note*. Descriptive variables were dummy coded for the logistic regression and ease of interpretation for the ANCOVAs. Whilst there are some small differences noted between the analyses with and without controls, the control analyses should be interpreted carefully due to unequal comparison groups, less power, missing data, and dummy coding (which may miss nuances) For a list of how the items were measured refer to OSF: <https://bit.ly/44V43DH>.

^ Employment Status (0 = Full-time, 1 = Part-time), Ethnicity (0 = White, 1 = People of Color), Employment Sector (0 = Female-dominated, 1 = Male-dominated), Sexual Harassment Experience (0 = Yes, 1 = No), Sexual Harassment Training (0 = Yes, 1 = No), Country of Residence (0 = Australia, 1 = United Kingdom).

**SOM Table 02**

|  | *df* | *F* | *p* | Partial *η*^2^ |
| --- | --- | --- | --- | --- |
| Corrected Model | 1, 1216 | 10.84** | < .001 | .22 |
| Response | 1, 1216 | 50.56** | < .001 | .04 |
| Type | 4, 1216 | 45.54** | < .001 | .13 |
| Gender | 1, 1216 | 22.89** | < .001 | .02 |
| Response*Type | 4, 1216 | 14.74** | < .001 | .05 |
| Response*Gender | 1, 1216 | 0.77 | .380 | .00 |
| Type*Gender | 4, 1216 | 2.53* | .039 | .00 |
| Response*Type*Gender | 4, 1216 | 0.31 | .875 | .00 |
| Occupation Time | 1, 1216 | 0.01 | .905 | .00 |
| Organization Time | 1, 1216 | 0.15 | .702 | .00 |
| Company Size | 1, 1216 | 1.60 | .206 | .00 |
| Employment Status^ | 1, 1216 | 2.62 | .106 | .00 |
| Ethnicity^ | 1, 1216 | 3.67 | .056 | .00 |
| Employment Sector^ | 1, 1216 | 1.51 | .220 | .00 |
| Sexual Harassment Experience^ | 1, 1216 | 0.17 | .677 | .00 |
| Sexual Harassment Training^ | 1, 1216 | 0.86 | .353 | .00 |
| Workgroup Gender Ratio | 1, 1216 | 0.01 | .915 | .00 |
| Country of Residence^ | 1, 1216 | 0.01 | .908 | .00 |
| Age | 1, 1216 | 0.17 | .681 | .00 |
| Job Level | 1, 1216 | 6.46* | .011 | .00 |
| Education | 1, 1216 | 0.43 | .511 | .00 |
| *Note*. ^ = dummy coded; **p* < .05; ***p* <.001 | | | | |

*ANCOVA Statistics for Response, Type, and Gender on Perpetrator Behaviour Appropriateness*

Two additional small effects were observed. First for job level, where higher job levels were associated with lower ratings of inappropriateness. Second, an interaction between type and gender, where male participants rated in particular the less severe behavior (gender harassment and seductive behavior) as less inappropriate than female participants as per Bonferroni adjusted post hoc comparisons (*p*s < .001).

**SOM Table 03**

*Coefficients of the Sexual Harassment Labeling Model with Controls*

|  |  |  |  |  |  |  | 95% CI for OR | |
| --- | --- | --- | --- | --- | --- | --- | --- | --- |
| Variables | | β | SE | Wald | *p* | OR | Lower | Upper |
| Participant Gender (Female) | |  |  |  |  |  |  |  |
|  | Male | -0.79 | .22 | 13.21 | < .001 | 0.45 | 0.30 | 0.70 |
| Sexual Harassment Type (Gender Harassment) | |  |  |  |  |  |  |  |
|  | Seductive Behaviour | 0.37 | .22 | 2.95 | .086 | 1.45 | 0.95 | 2.21 |
|  | Sexual Bribery | 2.99 | .40 | 56.04 | < .001 | 19.80 | 9.06 | 43.25 |
|  | Sexual Coercion | 3.47 | .49 | 51.38 | < .001 | 32.43 | 12.52 | 83.96 |
|  | Sexual Imposition | 2.78 | .35 | 62.31 | < .001 | 16.18 | 8.11 | 32.31 |
| Target Response (Not Interested) | |  |  |  |  |  |  |  |
|  | Interested | -1.47 | .21 | 50.79 | < .001 | 0.23 | 0.15 | 0.35 |
| Occupation Time | | -0.00 | .01 | 0.01 | .935 | 1.00 | 0.97 | 1.03 |
| Organization Time | | -0.03 | .02 | 2.38 | .123 | 0.97 | 0.94 | 1.01 |
| Company Size | | -0.05 | .07 | 0.45 | .505 | 0.95 | 0.83 | 1.10 |
| Employment Status^ | | -0.05 | .26 | 0.03 | .853 | 0.95 | 0.58 | 1.57 |
| Ethnicity^ | | -0.31 | .27 | 1.34 | .246 | 0.73 | 0.43 | 1.24 |
| Employment Sector^ | | 0.19 | .20 | 0.83 | .363 | 1.20 | 0.81 | 1.80 |
| Sexual Harassment Experience^ | | -0.48 | .26 | 3.48 | .062 | 0.62 | 0.38 | 1.02 |
| Sexual Harassment Training^ | | -0.35 | .21 | 2.84 | .092 | 0.70 | 0.47 | 1.06 |
| Workgroup Gender Ratio | | -0.00 | .00 | 0.17 | .685 | 1.00 | 0.99 | 1.00 |
| Country of Residence^ | | -0.30 | .47 | 0.39 | .532 | 0.74 | 0.30 | 1.88 |
| Age | | 0.01 | .01 | 0.47 | .492 | 1.01 | 0.99 | 1.03 |
| Job Level | | 0.07 | .08 | 0.77 | .380 | 1.07 | 0.92 | 1.26 |
| Education | | 0.08 | .07 | 1.19 | .276 | 1.08 | 0.94 | 1.25 |
| Constant | | 2.38 | .88 | 7.16 | .007 | 10.76 |  |  |

*Note*. OR = Odds Ratio; CI = confidence interval. The reference category for each variable is indicated in the parentheses next to the variable name. ^ indicates dummy coded and specified in the above information.

The effect of seductive behavior that is present in the main analysis was not observed.

**SOM Table 04**

*ANCOVA Statistics for Response, Type, and Gender on Confidence in Sexual Harassment Labeling Decision*

|  | *df* | *F* | *p* | Partial *η*^2^ |
| --- | --- | --- | --- | --- |
| Corrected Model | 1, 1207 | 12.41** | < .001 | .25 |
| Response | 1, 1207 | 63.43** | < .001 | .05 |
| Type | 4, 1207 | 64.73** | < .001 | .18 |
| Gender | 1, 1207 | 0.26 | .614 | .00 |
| Response*Type | 4, 1207 | 6.37** | < .001 | .02 |
| Response*Gender | 1, 1207 | 0.24 | .628 | .00 |
| Type*Gender | 4, 1207 | 0.78 | .537 | .00 |
| Response*Type*Gender | 4, 1207 | 0.90 | .463 | .00 |
| Occupation Time | 1, 1207 | 0.00 | .987 | .00 |
| Organization Time | 1, 1207 | 0.28 | .599 | .00 |
| Company Size | 1, 1207 | 0.42 | .518 | .00 |
| Employment Status^ | 1, 1207 | 0.00 | .970 | .00 |
| Ethnicity^ | 1, 1207 | 2.69 | .101 | .00 |
| Employment Sector^ | 1, 1207 | 1.37 | .243 | .00 |
| Sexual Harassment Experience^ | 1, 1207 | 9.94* | .002 | .00 |
| Sexual Harassment Training^ | 1, 1207 | 10.28* | .001 | .00 |
| Workgroup Gender Ratio | 1, 1207 | 1.53 | .216 | .00 |
| Country of Residence^ | 1, 1207 | 0.04 | .834 | .00 |
| Age | 1, 1207 | 0.27 | .600 | .00 |
| Job Level | 1, 1207 | 5.83* | .016 | .00 |
| Education | 1, 1207 | 0.53 | .468 | .00 |
| *Note*. ^ = dummy coded; **p* < .05; ***p* <.001 | | | | |

Three additional small effects were observed. First for job level, where higher job levels were associated with greater confidence in the labeling decision. Second, for sexual harassment experience where those who had experienced it had greater confidence in the labeling decision. Finally, for sexual harassment training where those who had done workplace training had greater confidence in the labeling decision.

**SOM Table 05**

|  | *df* | *F* | *p* | Partial *η*^2^ |
| --- | --- | --- | --- | --- |
| Corrected Model | 1, 1216 | 63.65** | < .001 | .63 |
| Response | 1, 1216 | 1,519.62** | < .001 | .56 |
| Type | 4, 1216 | 35.22** | < .001 | .10 |
| Gender | 1, 1216 | 0.62 | .430 | .00 |
| Response*Type | 4, 1216 | 74.34** | < .001 | .20 |
| Response*Gender | 1, 1216 | 4.56* | .033 | .00 |
| Type*Gender | 4, 1216 | 1.56 | .183 | .00 |
| Response*Type*Gender | 4, 1216 | 1.76 | .135 | .00 |
| Occupation Time | 1, 1216 | 0.15 | .702 | .00 |
| Organization Time | 1, 1216 | 2.79 | .095 | .00 |
| Company Size | 1, 1216 | 2.42 | .120 | .00 |
| Employment Status^ | 1, 1216 | 0.43 | .511 | .00 |
| Ethnicity^ | 1, 1216 | 1.49 | .223 | .00 |
| Employment Sector^ | 1, 1216 | 0.03 | .855 | .00 |
| Sexual Harassment Experience^ | 1, 1216 | 0.17 | .681 | .00 |
| Sexual Harassment Training^ | 1, 1216 | 0.33 | .564 | .00 |
| Workgroup Gender Ratio | 1, 1216 | 0.00 | .999 | .00 |
| Country of Residence^ | 1, 1216 | 1.51 | .219 | .00 |
| Age | 1, 1216 | 3.41 | .065 | .00 |
| Job Level | 1, 1216 | 1.04 | .307 | .00 |
| Education | 1, 1216 | 1.49 | .223 | .00 |
| *Note*. ^ = dummy coded; **p* < .05; ***p* <.001 | | | | |

*ANCOVA Statistics for Response, Type, and Gender on Target Behaviour Appropriateness*

The interaction between type and gender that is present in the main analysis was not observed.

**Power Calculation**

For the main analyses, a post-hoc power analysis using G*Power 3.1 (Faul et al., 2009) showed a sample size of 1,700 with an α = .05 level of significance achieved 80% power to detect a small effect size of *f* = .10.
